# Nonlinear rheological characteristics of single species bacterial biofilms

**DOI:** 10.1038/s41522-020-0126-1

**Published:** 2020-04-14

**Authors:** Saikat Jana, Samuel G. V. Charlton, Lucy E. Eland, J. Grant Burgess, Anil Wipat, Thomas P. Curtis, Jinju Chen

**Affiliations:** 1 School of Biomedical Sciences, University of Leeds, Leeds, UK; 2 School of Engineering, Newcastle University, Newcastle Upon Tyne, UK; 3 Interdisciplinary Computing & Complex BioSystems Research Group, School of Computing, Newcastle University, Newcastle upon Tyne, UK; 4 School of Natural & Environmental Sciences, Newcastle University, Newcastle upon Tyne, UK

**Keywords:** Biofilms, Environmental microbiology

## Abstract

Bacterial biofilms in natural and artificial environments perform a wide array of beneficial or detrimental functions and exhibit resistance to physical as well as chemical perturbations. In dynamic environments, where periodic or aperiodic flows over surfaces are involved, biofilms can be subjected to large shear forces. The ability to withstand these forces, which is often attributed to the resilience of the extracellular matrix. This attribute of the extracellular matrix is referred to as viscoelasticity and is a result of self-assembly and cross-linking of multiple polymeric components that are secreted by the microbes. We aim to understand the viscoelastic characteristic of biofilms subjected to large shear forces by performing Large Amplitude Oscillatory Shear (LAOS) experiments on four species of bacterial biofilms: *Bacillus subtilis*, *Comamonas denitrificans*, *Pseudomonas fluorescens* and *Pseudomonas aeruginosa*. We find that nonlinear viscoelastic measures such as intracycle strain stiffening and intracycle shear thickening for each of the tested species, exhibit subtle or distinct differences in the plot of strain amplitude versus frequency (Pipkin diagram). The biofilms also exhibit variability in the onset of nonlinear behaviour and energy dissipation characteristics, which could be a result of heterogeneity of the extracellular matrix constituents of the different biofilms. The results provide insight into the nonlinear rheological behaviour of biofilms as they are subjected to large strains or strain rates; a situation that is commonly encountered in nature, but rarely investigated.

## Introduction

Bacterial biofilms occur in diverse environments, such as aquifers^1^, rivers^2,3^, hydrothermal springs^4^, within sewer pipelines^5^, in bioremediation plants^6^ and various other places. Within these environments the biofilms can be subjected to extreme temperatures^4,7^, variation in physical forces^8^, changes in chemical concentrations^9^, changes in salinity^10^ and pH^11^. These extreme conditions impact a biofilm’s lifecycle, and yet, they are able to thrive by colonising a variety of surfaces. The ability of biofilms to withstand the above-mentioned dynamic environments is often attributed to the extracellular matrix (ECM),^12,13^ which is commonly described as a network of polymers, including extracellular polysaccharides (EPS), extracellular DNA (eDNA), proteins and various other components^14^. The ECM holds the polymeric constituents and bacterial cells together, thereby conferring the biofilm its rigidity and viscosity. In addition, the ECM is also known to perform a wide array of additional functions: such as acting as a reservoir of metabolites and signalling molecules^15^, offering decreased permeability to invaders or chemicals^16^ and as a promoter of virulence of microbes^17^. Despite its multifunctional nature, the ECM is often dubbed as the ‘dark matter’ owing to the limited information about its composition and the organisation of the polymers that constitute it^14^. Since the polymeric composition and its organisation within the ECM dictates the rheological behaviour of the biofilms (in addition to other functionalities); it becomes important to investigate the matrix viscoelasticity^17–19^. This could allow one to understand the role of biopolymers not only in conferring structure to the biofilm, but also, its biological and environmental functionalities.

Biofilms exhibit contrasting mechanical behaviour during their lifecycle: in early stages of growth in liquid cultures, cells become connected through the ECM and behave like viscoelastic liquid^20^. While, after growth on surfaces they can exhibit rheological characteristics similar to that of viscoelastic solids or liquids, which is dependent on a variety of environmental and physical factors. To study such complex rheological responses, a wide array of mechanical and spectroscopic techniques have been developed at length scales ranging from nanometers to a few millimetres^21–23^. Examples of such techniques include particle tracking rheology^24–26^, diffusing wave spectroscopy^27^, Brillouin-Raman microscopy^28–30^ and optical tweezing^31^. These techniques have been employed to quantify the structural heterogeneity within biofilm matrices at the scales of few microns, that arise due to changes in chemical environment^32^ or changes arising from modification of the genetic characteristics^18^. Rheometers are the most commonly used instrument for investigating viscoelasticity of biofilms and provides bulk characteristics (at millimeter scale) by performing dynamic oscillatory measurements^22^. Tests including amplitude/frequency sweep or creep and relaxation^33–36^ are the most commonly employed techniques. These measurement schemes have allowed investigators to shed light on: the functionality of eDNA in controlling the relaxation times^37^, the effect of chemical treatments on the viscoelastic moduli^38,39^ and the polysaccharide production mediated alteration in the mechanical toughness of biofilms^19^ and various other effects. Interfacial rheometry^40–42^, extensional measurements^43^, micro-cantilever^44^ and bioreactors interfaced with rheometers^45^ have allowed the growth of biofilm and pellicles under dynamic conditions; thereby allowing one to study the effects of dynamic conditions on the biofilm structure and variation in rheological properties. In most of these situations the strain applied to the biofilm is within the linear viscoelastic regime, as a result the biofilm structure remains intact. However, in realistic situations like in rivers, on ship hulls and in bioreactors the shear forces and rates near solid walls can be extremely high; which can induce large deformations within the biofilm network, thereby disrupting the structure of the underlying polymeric network. Such large strain or strain rates can result in local stiffening or softening, or appearance of significantly different material characteristics, which are rarely explored in case of biofilms^42^.

Large amplitude oscillatory shear (LAOS) is a type of dynamic oscillatory test performed on a strain-controlled rheometer, which involves measuring material response (stress) to increasing values of imposed oscillatory strain^46,47^. With the increasing strain amplitudes, the material response transitions from exhibiting a sinusoidal (linear) stress waveform to a non-sinusoidal (nonlinear) stress waveform^48^. By decomposing the nonlinear stress waveforms based on symmetry arguments^49^ and using Chebyshev polynomial analysis^50^ one can analyse the contribution of higher order harmonics to gain invaluable insights. Material descriptors like intracycle strain stiffening (or softening) and intracycle shear thickening (or thinning) indices can reveal a unique fingerprint of the material^50^. Owing to higher sensitivity, LAOS also finds application in detection of architecture and branching characteristics of polymer melts^51–53^. In the past decade, LAOS has become a canonical technique for quantifying the rheological characteristics of polymers, soft solids, gels, emulsions and various other complex fluids and materials^48,54^. The techniques have also been successfully applied to understand the intracycle strain dependent hardening/softening or thickening/thinning of biological materials, examples of which include: mucus of the gastropod^55^, hagfish slime^56^, fibrobalst cells^57^, fibrin/collagen gels^58^, vocal fold tissues^59^, pluronic/hyaluronic acid^60,61^, Xanthan gum^62^ and blood^63^. For biofilms, LAOS provides us with the ability to determine the nonlinear material response of biofilms when subjected to large shear. In addition, one might also be able to perform genetic manipulation to control polymer production in biofilms and study how the interactions of the polymers play a role in shaping the rigidity and viscosity or other biological functionalities of biofilms.

**Fig. 1:**
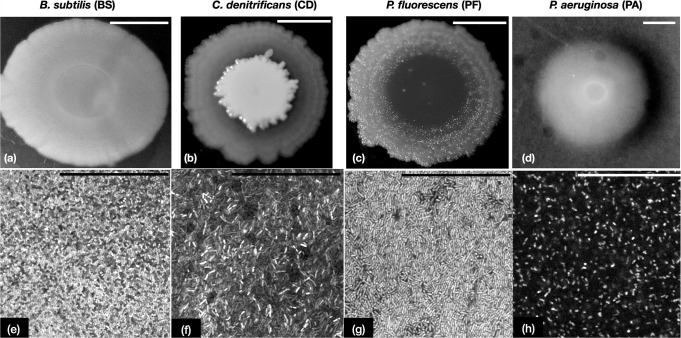
Figure shows the macroscopic and microscopic pictures of biofilms. **a**–**d** Panels show representative pictures of single species biofilms spreading out radially from a 2 μL droplet on the agar plates for a period of 6 days. Scale bars for panels **a**–**d** are 5 mm. **e**–**h** Panels show the distribution of cells in scraped off biofilms as seen under a confocal microscope. The biofilm scrapings were taken from thick mat of biofilm that was formed on agar plates after a growth period of 3 days. Scale bars for panels **e**–**h** are 50 μm.

In this paper we employ a combination of small and large amplitude oscillatory shear (SAOS and LAOS) tests to characterise the rheology of four species of bacterial biofilms: *Bacillus subtilis* (BS), *Comamonas denitrificans* (CD), *Pseudomonas fluorescens* (PF) and *Pseudomonas aeruginosa* (PA). Biofilms from these microbes tend to spread out radially into circle shaped colonies as seen in Fig. 1 (a–d) when a droplet of bacteria culture deposited on an agar plate. Whereas if the microbes are smeared uniformly on the agar plates, they form a continuous mat like biofilm. Scrapings of these mat like biofilms exhibit contrasting differences in cellular packing under a confocal microscope, which can be seen in Fig. 1 (e–h). Small amplitude oscillatory tests reveal that the linear viscoelastic moduli and yield stresses of the single species biofilms vary by orders of magnitude. Furthermore, by performing LAOS experiments and using Chebyshev polynomial analysis we are able to capture the subtle as well as distinct changes in the intracycle strain stiffening and shear thickening characteristics of the biofilms in the Pipkin diagram. The biofilms also exhibit differences in transition to nonlinear viscoelastic behaviour and energy dissipation characteristics, indicating that polymeric composition can play a role in dictating such nonlinear behaviours. Together, these results show that LAOS can be a useful tool in characterising the nonlinear rheological behaviour in biofilms of different species.

## Results

The following sections describes our findings on the linear and nonlinear rheological characteristics of the different species of biofilms. We first perform the linear viscoelastic tests to quantify the differences in the single species biofilms.

### Linear viscoelastic moduli varies across single species biofilms

**Fig. 2:**
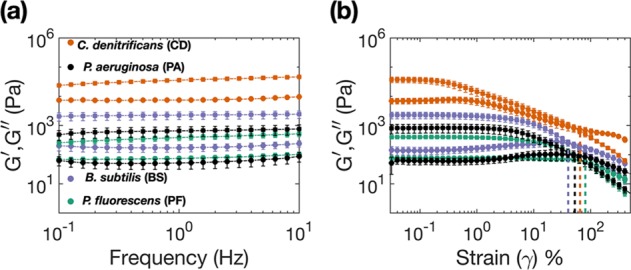
Plot showing the elastic (${G}^{\prime}$, denoted by squares) and viscous moduli (*G″*, denoted by circles) of different biofilms: *Bacillus subtilis* (BS), *Comamonas denitrificans* (CD), *Pseudomonas fluorescens* (PF) and *Pseudomonas aeruginosa* (PA). **a** Shows the variation in the elastic and viscous moduli as a function of a frequency, also known as frequency sweep. **b** Shows the log–log plot of the elastic and viscous moduli as a function of applied strain, also known as the amplitude sweep. (*n* ≥ 5, all error-bars correspond to one standard deviation).

Figure 2 (a) shows the frequency sweep results for the different species of biofilms. *Pseudomonas fluorescens* biofilms exhibit the lowest elastic moduli amongst the tested species while *Pseudomonas aeruginosa* exhibited the minimum viscous modulus. The elastic (${G}^{\prime}$) and viscous moduli (*G″*) of *P. fluorescens* measures 385*Pa* and 75*Pa* at 1 Hz, while the measurements for *P. aeruginosa* was measured to be 655*Pa* and 52*Pa* at 1 Hz, respectively. In contrast, for *Comamonas denitrificans* the elastic (${G}^{\prime}$) and viscous moduli (*G″*) measured 35770 Pa and 7400 Pa at 1 Hz, which are higher by a factor of ~10^2^ when compared to *P. fluorescens*. The moduli for *Bacillus subtilis* are in the intermediary range; ${G}^{\prime}$ ~2250 Pa and *G″* ~172 Pa at 1 Hz. For all the species of biofilms the moduli exhibits a plateau up to 10 Hz. Beyond 10 Hz the inertia of the instrument dominates and therefore the data have been discarded.

Figure 2 (b) shows the results from oscillatory amplitude sweep for the four different species of biofilms that were carried out at 1 Hz for strain amplitudes ranging from 0.025–400%. It is evident from the plot that the linear viscoelastic regime, which is denoted by the plateau of ${G}^{\prime}$ in the amplitude sweep plot varies for the different biofilms. *P fluorescens* exhibits linear behaviour for strain amplitudes (*γ* < 10%), *P. aeruginosa* for (*γ* < 4%), *B. subtilis* for (*γ* < 5%) and *C. denitrificans* for (*γ* < 0.5%). Beyond the linear viscoelastic regime the elastic moduli continuously decreases for increasing strain amplitudes and exhibits a consistent decay, indicating a global strain softening behaviour up to a strain amplitude of 500%. The viscous modulus for *B. subtilis*, *C. denitrificans* and *P. aeruginosa* show distinct humps at *γ* = 30%, *γ* = 0.7% and *γ* = 25%, which are indicative of Type-III weak strain overshoot. Such behaviour is commonly seen in soft glassy materials and can arise due to structural rearrangements arising within the biofilm^51^. A closer look at the viscous modulus component of the amplitude sweep for CD shows a two step yielding signature at 0.7% and 200% strain, similar to that observed in colloidal systems^64–66^, which hypothesises successive bond and cage breaking at two separate strain amplitudes. However, such phenomena for biofilms are yet to be quantified and physically understood in terms of the underlying structure and the matrix constituents. The single species biofilms also show distinct crossover points for elastic and viscous modulus in the amplitude sweep plot. The corresponding yield stresses (*σ*_*Y*_) for BS, CD, PF and PA are found to be 248 Pa, 822 Pa, 57 Pa and 100 Pa, respectively.

### Intracyle strain stiffening/softening characteristics shows subtle differences amongst biofilm of different species

**Fig. 3:**
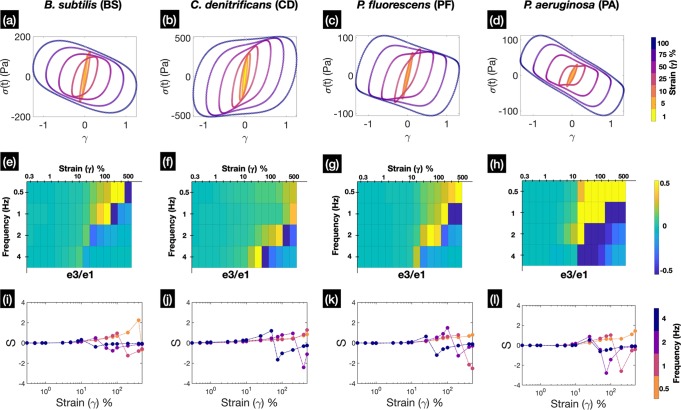
Figure shows the intracycle strain hardening/softening characteristics of biofilms. Plots **a**, **b**, **c**, **d** show the representative elastic Lissajous-bowditch plots for the different species for strain amplitudes ranging from 1–100% at oscillatory frequency of 2 Hz. Figures **e**, **f**, **g**, **h** show the heat maps of the normalized value of *e*_3_ with respect to *e*_1_, which show the distinct regions in the Pipkin space where strain hardening/softening occur (*n* ≥ 5, for standard deviation please see Supplementary Fig. 1). Figures **i**, **j**, **k**, **l** show the value of the strain stiffening index *S* at different frequencies for the different species of biofilms that were tested (*n* ≥ 5, for standard deviation please see Supplementary Fig. 2).

We subsequently turn our attention to the behaviour of the bacterial biofilms when they are subjected to large strain amplitudes. The raw waveforms, which are recorded by the rheometer as numerical values of stress versus time and strain versus time, can also be parametrically represented as stress versus strain plots. These plots are commonly known as elastic Lissajous Bowditch (LB) plots and provide a geometrical description of the state of the material. In the linear viscoelastic regime, the LB plots takes the shape of a prolate ellipse; but the shape becomes progressively distorted into parallelogram like shapes as the material is subjected to higher values of strain amplitudes and the behaviour of the biofilm transitions to the nonlinear regime. Figure 3 (a–d) shows the elastic Lissajous-bowditch plots for each of the tested strains of single species biofilms at 2 Hz for strain amplitudes ranging from 1–100%. One can observe from Fig. 3 (a–d) that elastic LB plot for each species depicts a distinct shape and size for a given strain amplitude. The maximum total stress seen in the LB plots for CD biofilms is ~500 Pa, which is about five times higher as compared to PF or PA biofilms ~100 Pa; while for BS the maximum value of the stress is ~180 Pa. Occurrence of parallelogram like shapes are indicative of plastic flow in the biofilms and changes in the slope of major axis of the parallelogram refers to the presence of strong nonlinearities in the material.

A more quantitative description of the LB plots can be obtained by calculating the third (*e*_3_) and the first (*e*_1_) order Chebyshev polynomial coefficients using MITlaos software. The third order coefficient of Chebyshev polynomial aids in a physical interpretation, a positive or negative value denotes intracycle strain hardening or softening, respectively; and gives us a measure of elastic nonlinearities in the material. Stiffening can be understood as sudden increase in stress due to increasing values of applied strain, while softening represents a decrease in the value of stress due to increase in the applied strain. Depending on the polymeric composition of the matrix and the interactions between the constituent polymers, stiffening or softening can occur at different points in the Pipkin space. Figure 3 (e–h) shows a plot of the ratio of *e*_3_∕*e*_1_ at various points in the Pipkin space for the different species biofilms (for standard deviation of *e*_3_∕*e*_1_ see Supplementary Fig. 1). We find that PF biofilms shows strain stiffening characteristics at 0.5 Hz and 1 Hz, with the maximum values of *e*_3_∕*e*_1_ occurring in between strain amplitudes 200–500% and 100–200%, respectively. Beyond strain amplitude values of 200% at 1 Hz, the PF biofilms soften. BS biofilms show similar strain stiffening characteristics when compared to PF biofilms at both 0.5 Hz and 1 Hz. Strain stiffening for BS biofilms occur at a slightly smaller strain amplitude (75–400% at 0.5 Hz and 50–100% at 1 Hz) and the softening also happens only slightly earlier compared to biofilms of PF. CD shows strain stiffening at 0.5 Hz and 1 Hz for all values of strain amplitudes and doesn’t show any softening behaviour at this particular frequencies. The peak values of strain stiffening for CD occurs at 2 Hz and 4 Hz, at strain amplitudes of 200% and 50%, respectively, followed by softening and recovery. Similar to CD biofilms the peak values occur for PF biofilms at 75% and 25% strain amplitude at 2 Hz and 4 Hz. In contrast, BS biofilms only show slight evidence of strain stiffening at 2 and 4 Hz. PA biofilms exhibit persistent strain stiffening which starts at 25% strain amplitude at 0.5 Hz and lasts up to 50%. Similarly, at frequencies of 1 Hz and 2 Hz, strain stiffening initiates at 10% strain amplitude and persists upto 100% and 25% respectively. The softening beyond these values of strain amplitudes is indicative of breakdown of the underlying structure of the PA biofilms after which it softens rapidly. At a frequency of 4 Hz, the PA biofilms soften at strain amplitude of 25% and subsequently shows recovery of the structure as the value *e*_3_∕*e*_1_ increases. The stiffening behaviour of PF and PA biofilms which belong to the same genus, differ in certain aspects: Firstly, the onset of stiffening is much quicker for PA biofilms which is evidenced by pipkin diagrams 3 (b3–b4). Secondly the softening or breakdown of structure is more extensive in PA biofilms and it takes longer to recover when compared to PF biofilms.

We also present the results of strain stiffening index (*S*) for the different biofilms in Fig. 3 (i–l), which is defined by equation (4) (for standard deviation of *S* see Supplementary Fig. 2). A value of *S* > 0 indicates intracycle strain stiffening behaviour while *S* < 0 represents strain softening behaviour. In the Pipkin space, all four species of biofilms show evidence of strain stiffening at 0.5 Hz for strain amplitudes <500%. As the frequency of oscillation increased, the transition from stiffening to softening occurs at progressively smaller strain amplitudes for all the biofilms. The largest value of strain stiffening index at 0.5 Hz occurs for BS biofilms (*S* ~ 2), while for CD, PF and PA biofilms, the indices show a much lower value. At 2 Hz, CD, PF and PA biofilms shows persistent stiffening until strain amplitudes of 200%, 100% and 25%, respectively. The stiffening indices reach a maximum value of *S* ~ 1, *S* ~ 2 and *S* ~ 1, respectively, for CD, PF and PA biofilms. BS biofilms at 2 Hz mildly strain stiffen at intermediate strain amplitudes upto 25% and 50% and subsequently exhibit strain softening. At 4 Hz, for BS, PF and PA biofilms the strain stiffening index is minimal for all values of strain amplitudes, while CD biofilms are found to exhibit the maximum value of the stiffening index at strain amplitude of 50%.

### Intracyle shear thickening/thinning characteristics shows distinct differences amongst biofilm of different species

**Fig. 4:**
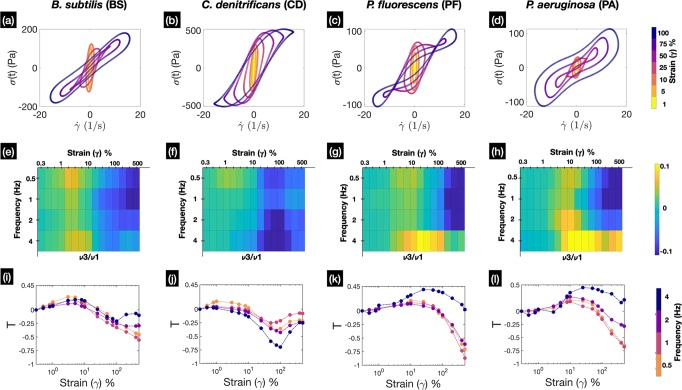
Figure shows the intracycle shear thickening/thinning characteristics of biofilms. Plots **a**, **b**, **c**, **d** show the representative viscous Lissajous-bowditch plots of the different species for strain amplitudes varying from 1–100% at frequency of 2 Hz. Figures **e**, **f**, **g**, **h** show the heat maps of the normalized value of *ν*_3_ with respect to *ν*_1_, which indicates the distinct regions in the Pipkin space where shear thickening/thinning occur (*n* ≥ 5, for standard deviation please see Supplementary Fig. 1). Figures **i**, **j**, **k**, **l** show the value of the shear thickening index *T* at different frequencies for the different species of biofilms that were tested (*n* ≥ 5, for standard deviation please see Supplementary Fig. 2).

To investigate the intracycle viscous nonlinearities occurring in the biofilms we turn our focus to the viscous Lissajous Bowditch plots, which show the variation of stress (*σ*) with strain rate ($\dot{\gamma }$). Akin to elastic LB plots, the viscous LB plots can be constructed by parametrically eliminating time, from the time series signals of stress and strain rates. The viscous LB plots describe the viscous response of the material through a graphical representation. Figure 4 (a–d) shows the viscous LB plots for the different species of biofilms at an oscillatory frequency of 2 Hz and for strain amplitudes ranging from 1 to 100%. At small values of strain amplitudes, the LB plots takes a circular shape representing the expected behaviour in the linear viscoelastic regime. However, as the biofilm is subjected to increasing values of strain amplitudes, the circle gets distorted to different shapes indicating the presence of nonlinearities in the material. For CD biofilms the viscous LB plots resemble sigmoids (Fig. 4 (b)), indicating the existence of strain stiffening or softening behaviour. For both PF and PA biofilms, the material exhibits large nonlinearities at certain intermediate and high values of strain amplitudes and one also observes the appearance of self-intersecting loops (Fig. 4 (c–d)). These secondary loops are indicative of a phenomena known as stress overshoot, where reversible structural breakdown of the material occurs^67^.

While the viscous LB plots aids in qualitative description of the material, a more quantitative description of the nonlinearities can be gained by plotting the ratio of third order to first order Chebyshev coefficients (*ν*_3_∕*ν*_1_) in the Pipkin space. A positive value of *ν*_3_ indicates intracycle shear thickening while a negative value indicates shear thinning. Thickening can be understood as sudden increase in stress as function of increasing strain rate, while thinning represents a decrease in the value of stress due to an increase in the applied rate. It is evident from Fig. 4 (e–h), that each of the tested biofilms shows a varying degree of viscous nonlinearities in the Pipkin space (for standard deviation of *ν*_3_∕*ν*_1_ see Supplementary Fig. 1). For all the tested frequencies, BS biofilms exhibit slight intracycle thickening at amplitudes below 25%, beyond which they show thinning behaviour. The maximum thickening for BS biofilms occurs at 0.5 Hz and 4 Hz for strain amplitudes of 3–5% and 5–10%, respectively. While, the minimum values of thickening for BS biofilms is seen at 0.5 and 1 Hz, for strain amplitudes ranging from 400 to 500%. For PF biofilms, shear thinning occurs at 0.5, 1 and 2 Hz, for strain amplitudes ranging from 200 to 500%. PF biofilms also show mild strain stiffening for strain amplitudes ranging from 3 to 10% at frequencies below 4 Hz, thereby showing a behaviour similar to BS biofilms. However, the maximum thickening for PF occurs at 4 Hz in between strain amplitudes of 10–100% and the biofilm doesn’t show any thinning behaviour at the given frequency. This is quite different to the behaviour of BS biofilms at 4 Hz for which thinning starts for strain values >25%. PA biofilms exhibit a behaviour similar to that of PF biofilms at both 0.5 Hz and 4 Hz. For strain amplitudes >100% at 4 Hz magnitudes of ratio of thickening indices is larger when compared to biofilms of PF. At 2 Hz and for strain amplitudes ranging from 5 to 25% the magnitude of thickening indices are higher when compared to that of PF (or BS). CD biofilms exhibit the most contrasting behaviour amongst the tested species, showing only a slight value of shear thickening across the Pipkin space. The peak of the thickening behaviour for CD occurs at 0.5 Hz, in between strain amplitudes of 0.8–8%. The minimum value of thickening for CD occurs at 2 and 4 Hz, in between strain amplitudes of 50–200%.

We also present the plot of thickening index (*T*) in Fig. 4 (i–l) for the four species of biofilms that we had investigated (for standard deviation of *T* see Supplementary Fig. 2). A value of *T* > 0 indicates intracycle shear thickening behaviour, while *T* < 0 represents shear thinning behaviour. At 4 Hz, thickening is the dominant behaviour for both BS, PF and PA biofilms, though it occurs at different strain amplitudes for the different species. For BS biofilms, thickening index shows a negative value for stain amplitude of 75%, in contrast PA and PF show persistently positive values of *T* for strain amplitudes >5% at 4 Hz. The maximum value of the thickening index (amongst all species) *T* ~ 0.5 is found to occur for PA biofilms at 4 Hz. CD exhibits a contrasting behaviour compared to the other species as it shows a consistent thinning behaviour at 4 Hz. Thinning behaviour is also predominant at other frequencies and for strain amplitudes >10%. The minima of *T* for CD biofilms occurs close to 100% strain after which an increase in the values of *T* is seen for all the frequencies. The minimum value of *T* ~ − 0.75 for CD occurs at 4 Hz and strain amplitude of 100%, while the maximum value of *T* ~ 0.15 occurs at 0.5 Hz at 1% strain amplitude. At large values of strain amplitudes >100% and for all the tested frequencies, CD biofilms exhibit recovery of the network structure (demonstrated by increase of *T* after a minimum), which is sparingly observed for the biofilms of any other species. The negative values of *T* at specific strain amplitudes, could also be correlated to the two step yielding behaviour. The dip in *T* might be representative of the characteristic bond breaking behaviour seen in colloidal systems^64–66^ and could explain the presence of a two step hump as seen in the amplitude sweep (Fig. 2 (b)). All of these measures capture the variation in the intracycle viscous nonlinearities that occur in the different species.

### *C. denitrificans* biofilms exhibit highest energy dissipation

**Fig. 5:**
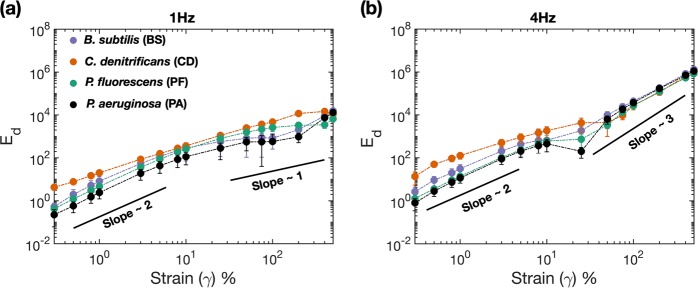
Figure shows the elastic energy dissipation characteristics for the different species of biofilms. Energy dissipation values measured **a** at 1 Hz frequency and **b** at 4 Hz; during the dynamic oscillatory testing (*n* ≥ 5, error-bars correspond to one standard deviation). Solid lines with indicative slopes are presented to show the approximate power law scaling between dissipated strain and magnitude of strain amplitude.

To gain insight into the energy dissipation characteristics of the biofilms, we numerically integrated the area enclosed by the elastic Lissajous plots. For a perfectly elastic material, the plot of stress vs. strain typically yields a straight line; indicating zero energy dissipation and a complete recovery of the material to its original state. However, for complex materials like biofilms and other soft solids the energy dissipated can be nonzero, indicating an incomplete recovery of material. Figure 5 (a) and (b) shows the energy dissipated by the various species of biofilms at 1 Hz and 4 Hz, respectively. Within the linear regime, the dissipated energy for all the species that were tested show quadratic dependence on the strain amplitude *γ*_*o*_, which confirms the analytic solution ${E}_{d}=\pi {\gamma }_{o}^{2}{G}_{1}^{^{\prime\prime} }$^50,68^. Amongst the different species the variation in energy dissipation is rather large; with PA showing the least amount of dissipation. BS is found to dissipate slightly more energy than PF, whereas dissipation by CD is an order of magnitude higher as compared to BS. The larger amount of dissipation indicates that CD is more prone to having irreversible structural changes as compared to any of the other biofilms. Amongst the same genus of *Pseudomonads*, *aeruginosa* exhibits comparatively better elastic recovery as compared to *fluorescens*, which points to the existence of biomolecules aiding in elastic recovery. Beyond the linear regime and at 1 Hz, ${E}_{d} \sim {\gamma }_{o}^{1}$; however across all values of strain amplitudes, CD consistently dissipates higher energy by at least half an order of magnitude when compared to all the other biofilms. At 4 Hz and for *γ*_0_ > 10%, *E*_*d*_ scales as ${\gamma }_{o}^{3}$; which possibly indicates failure of the material, allowing it to dissipate energy rapidly after the point of failure. Similar effects of material failure, on energy dissipation characteristics has been discussed in context of optical coatings by Chen et al.^69^.

### Onset of nonlinearity defined by Medium amplitude oscillatory shear region differs amongst species

**Fig. 6:**
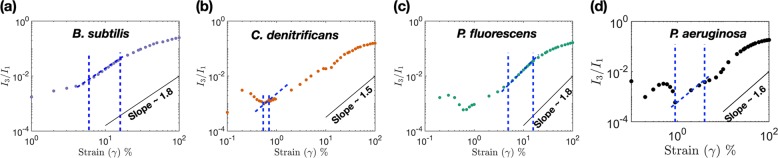
Figure shows the onset of nonlinearity in different species of biofilms. Plots show the variation in the ratio of third (*I*_3_) to first (*I*_1_) order harmonic as a function of strain amplitude for **a** *B. subtilis* **b** *C. denitrificans* **c** *P. fluorescens* and **d** *P. aeruginosa* at a frequency of 1 Hz. Lines with slopes of 1.8, 1.5, 1.8 and 1.6 are indicated as reference in the corresponding plots. Blue dotted lines denotes the MAOS region within which the lines of particular slope are observed.

In the linear regime, there is minimal distortion of the stress signal; as a result the first order harmonic signals dominate and contribution from higher order signals are minimal. As the strain amplitude on the biofilm sample is increased, the stress waveforms distort and contributions from higher order harmonics become significant. Increase in intensity of higher order harmonics (*I*_*n*_) provides one with an understanding of the nature of nonlinearity developing within the material. Normalized values of higher order harmonics (*I*_*n*_∕*I*_1_) can be obtained from the files that are output by MITlaos and contains information on fourier transform spectrum of stress. One way of exploring the onset of nonlinearity in the different species of biofilm is by comparing the intensity of third to first order harmonic (*I*_3_∕*I*_1_) as a function of the strain amplitude, at a constant frequency of 1 Hz. Similar tests undertaken on typical model polymers exhibit a scaling of ${I}_{3}/{I}_{1} \sim {\gamma }_{o}^{2}$. This particular region of quadratic scaling is known as Medium Amplitude Oscillatory Shear (MAOS) and denotes the transition from linear to nonlinear behaviour of the sample under consideration. Figures 6 (a), (b), (c) and (d) show the scaling relation, which is indicative of the onset of nonlinearity for the different species of biofilms. *B. subtilis* shows a scaling of 1.8 for strain amplitudes ranging between 6 and 12%, while *C. denitrificans* shows a scaling of ∝ 1.5 for strain amplitude ranging between 0.55 and 0.7%, *P. fluorescens* exhibits a scaling of ∝ 1.8 for strain amplitude from 8 to 13% and *P. aeruginosa* exhibits a scaling of ∝ 1.6 for strain amplitude from 0.9 to 4%. By comparing the MAOS regions of different microbial species, once comes to the conclusion that *C. denitrificans* starts exhibiting nonlinearity at very small strain amplitudes; but the rate of growth of nonlinearity is smaller when compared to any of the other species. For model polymeric systems, like linear polymers a scaling exponent of ~2 is usually observed; while a slope of ~1.8 has been observed for branched polymer systems^51,70^. Both *P. fluorescens* and *B. subtilis* show a scaling of 1.8 indicating a behaviour similar to that of branched polymer networks^51,52,70^. However, one should be careful about drawing such conclusions because biofilms are complex mixtures of multiple polymers. LAOS experiments that have found power law dependent scaling characteristics in MAOS region have only been performed for model and synthetic polymer systems.

## Discussion

We have investigated the rheological characteristics of four different species of bacterial biofilms in the linear viscoelastic and nonlinear regime. In the linear regime, biofilms show substantial differences in viscoelastic moduli and yield stresses that are orders of magnitude different from each other. The amplitude sweeps for most of the species exhibit a type III hump for the viscous modulus with increasing strain amplitude, which is reminiscent of the structural rearrangement that occur in soft colloidal systems. The frequency sweeps exhibit a weak power law pointing towards similarities between biofilms and colloidal gels. By subjecting the biofilms to increasing values of strain amplitudes at different frequencies we are able to capture the elastic and viscous nonlinearities occurring in the biofilms using the Pipkin diagram. The elastic nonlinearities show subtle variations in the phase space of frequency and strain amplitude (i.e. the Pipkin diagram), while the viscous nonlinearities show contrasting differences dependent on the species. The biofilms also exhibit large differences in the elastic energy dissipation characteristics indicating a varying amount of mechanical resilience of the extracellular matrix. By studying the slope of the third to first harmonic, we find that the onset of nonlinearity occurs much earlier in *C. denitrificans* when compared to the other species. The nonlinear parameter (*I*_3_∕*I*_1_) grows at a much higher rate for *B. subtilis* and *P. fluorescens* indicating a larger degree of nonlinearity of the ECM for these two species (albeit their linear viscoelastic moduli is significantly lower when compared to *C. denitrificans*).

Such contrast in the rheological measures of biofilms might be a result of a combination of factors. The first factor might be the way cells order and pack themselves in biofilms. As seen in Fig. 1(b2) the packing in CD biofilms is quite disordered and the disorder in packing extends to the out of focal plane. Whereas in PF the cells lie mostly within the focal plane but there is lot of spatial disorder. PA shows very sparse packing of cells, which can be seen in Fig. 1(b4) and cellular disorientation spans out of the focal plane, even greater than that observed for CD. The second factor is the extracellular polymers that constitute the ECM which are known to form links between cells and between the cells and the matrix providing the biofilm its rigidity and structure. Proteins like RbmA are known to control cell–cell connections and therefore determine the ordering of cells within *V. cholerae* a biofilm^71^. However, similar knowledge of the polymeric components of the matrix and their roles for the species that we have tested are still lacking. For example in *B. subtilis*, the matrix comprises of a large molecular weight exo-polysaccharide (EPS), whose exact composition remains yet undiscovered^72^. While, the major protein components are TasA which causes the rugosity of the biofilm colonies and BslA that controls the hydrophobicity. Minor protein component TapA is known to control the assembly of TasA fibres. However, the role of individual polymers in determining the rheology of the overall matrix in *B. subtilis* still continue to be investigated^73^. The matrix components of biofilms of *C. denitrificans* still remain unknown and have only been quantified in terms of mass fraction of proteins, nucleic acids and polysaccharides^74^. Rheological tests on this particular species of biofilms have not been performed to the best of our knowledge. In *P. fluorescens*, the biofilms consist of an acetylated form of cellulose fibre, together with lipo-polysachharides, fibrils and attachment factors like PNAG (poly-N-acetylglucosamine); which provide structural integrity to the wrinkly spreader biofilms^75,76^. Functional amyloids produced by the fap operon have been found to modulate the hydrophobicity of *P. fluorescens* biofilms and also confer mechanical strength^77^. Our knowledge of the matrix constituents of *P aeruginosa* is substantially advanced, as compared to other microbes owing to decades of research on the model organism. It is well known that the polysaccharide Pel acts as a scaffold for the biofilm and helps in maintaining the intercellular interactions^78^, while the polysaccharide component Psl initiates biofilm formation by modulating cell–cell and cell-surface attachment^79,80^. An over-expression of the polysachharide alginate results in a mucoid biofilm^19^. The protein CdrA has been found to control the cellular packing of cells and provide protection against proteolysis by interacting with Psl^81^. More recently the matrix protein LecB has been found to bind to Psl and helps in retention of cells and polysaccharides within the PA matrix^82^. This highlights the diversity of polymers in the various biofilms, however their exact contribution to rheology of biofilms and especially large strain behaviour needs to be carefully investigated.

In our experiments we scrape a uniform mat of biofilm and then use the pooled scrapings for rheological measurements. One of the major concerns is that the process of scraping can introduce defects or disrupt the structure of a continuous material, thereby affecting the rheological measurements. This is an plausible situation if the cultivated biofilms are very weak and especially for submerged biofilms. Since our biofilms are cultivated at the agar-water-air interface, they are more stiffer than their submerged counterparts. While scraping with a glass slide (imposing large strains), we find that the torn part of the biofilm mat retains its cohesion and tears off cleanly at the edges. This possibly indicates that the material still retains its continuous properties even after application of large strain (please see Supplementary Fig. 3, Supplementary note 1). Multiple instances of such scrapings with continuous material properties are pooled together to create a composite biofilm test sample, which is expected to exhibit rheological characteristics similar to the bulk material. In addition, biofilms are hypothesised to be thixotropic materials which means that their material structure and rheological properties can recover, even after the application of large strains. Such behaviour was recently confirmed by Yan et al. in *V. cholerae* biofilms^83^, where even after two cycles of amplitude sweep of up to 1000% strain; the linear viscoelastic measures were found to be similar to each other. Our experiments also show a similarity of elastic or viscous LB plots when the CD biofilms are subjected to LAOS sequences of up to 500% strain amplitude (please see Supplementary Fig. 4, Supplementary note 2).

While our study uses biofilm grown on agar plates which is an artificial system, in reality biofilms within natural environments are subjected to shear forces, varying amounts of hydration (water content), temperature and various other factors. Many of these factors including a variety of tools have confounded rheological measurements of biofilms in the past^84^, as describing the rheological state of a biofilm (akin to colloids) remains a challenging proposition. Part of the problem can be attributed to the active nature of biofilms. For example, as the biofilms start growing on surfaces, they secrete extracellular polymers and the concentration of the polymers depends on the water content of the system. However, as a biofilm’s growth progresses over time (given a fixed volume of water), the concentration of the polymeric substances would increase, which could result in transition from a dilute to a concentrated polymeric system, resulting in a different rheological state. Another complexity arises from the fact that extracellular polymers of biofilms are a mixture of multiple polymers; which could interact amongst themselves. In addition, shear forces play an implicit role in shaping the structure of biofilms, the constituent polymeric molecules in the matrix can stretch, reorienting the cells thereby causing local ordering^85,86^. Beyond a certain limit of shear forces, the structures can rupture forming clusters and the clusters can hydro-dynamically interact with each other. On a rheometer, such interactions can show up as two step yielding signatures, which we have observed in the case of *C. denitrificans*^64–66^. To summarise, in order to better characterise biofilm rheology a better description of the rheological state of the biofilm, which includes a description of constituent macromolecules and their interactions would be essential.

With advances in molecular microbiology, investigations involving the impact of biomolecules/biopolymers have led to interesting insights into a variety of biofilm functionalities. However, a similar knowledge of the molecular constituents and their effects on biofilm rheology is still limited and only spans a few species^17–19^. Biochemists in particular can isolate and help decipher the role of small molecules that cause short- and long-range interactions amongst the matrix constituents and the cells, which may cause stiffening/thickening of the matrix or various other effects. Alternatively, microbiologists can also engineer mutants that lack ability to express a particular biomolecule and investigate the absence of polymeric component on the rheological behaviour of the biofilm matrix, using LAOS. This could allow one to shed light on the role of the particular component in biofilm rheology. While we have described LAOS as a tool to mechanically explore biofilm rheology, it is by using tools from biochemistry/molecular microbiology; that the real potential of LAOS in deciphering the interactions of biopolymers can be realised. Advanced tools like confocal rheoscope^87^, small angle neutron scattering^88^, together with LAOS can help us visualise the cellular structure/orientation of cells and greatly enhance our knowledge of matrix viscoelasticity of biofilms.

Overall, our results provide insights into the nonlinearities occurring in biofilms at large shear forces, a situation that could be more prevalent than perceived. It also lays the groundwork for future investigations that could possibly use genetic manipulation to dissect the role of matrix polymers and their interactions, in altering matrix viscoelastic characteristics. By combining the above-mentioned array of interdisciplinary tools, we hope to continue to gain novel insights into the material characteristics of biofilms and their functionalities, in the near future.

## Methods

### Bacteria and biofilm growth conditions

*Pseudomonas fluorescens* is a gram-negative, multi-flagellate, obligate aerobe that was isolated from prefilter tanks in Sweden and can also be found acting as a biocontrol agent on the plant roots. *Bacillus subtilis* is a soil and gut dwelling, multi-flagellate, gram-positive bacterium and is a model system for studying various characteristics of biofilms. *Comamonas denitrificans* is a uniflagellate, gram-negative bacterium that plays a role in the denitrification process and is one of the dominant species found in the Birtley Wastewater Treatment plant (Northumbrian Water). *Pseudomonas aeruginosa* is an opportunistic pathogen that causes chronic infections in humans and is also commonly found in lungs of cystic fibrosis patients. Single species biofilms of *Bacillus subtilis* (*B. subtilis* 168), *Pseudomonas fluorescens* (DSMZ-50090) *Comamonas denitrificans* (DSMZ-17887) and *Pseudomonas aeruginosa* (DSMZ-22644) were grown overnight in LB Miller (Molecular Dimensions MD12-103, 25g/L), Nutrient (Sigma-Aldrich N7519, 8g/L), Tryptic Soy (Sigma-Aldrich 22092, 30g/L) and Nutrient (Sigma-Aldrich N7519, 8g/L) broth respectively. For imaging of radially spreading biofilms, a 2 μL droplet of bacteria culture is deposited on the respective agar plates and were stored at room temperature for six days. For all rheology experiments, a 150 μL droplet of the overnight culture was pipetted onto the respective 1.5% agar plates, respectively, and was smeared aseptically over the agar surface using a L shaped spreader. The inoculated bacteria were allowed to develop thick mat like continuous biofilms on the agar surfaces for 72 h (at room temperature) and were then harvested for experiments.

### Rheometry

For rheological experiments, mat like biofilms were gently scraped off from the agar plates using a glass slide and the aggregate is placed on the stage of a Malvern Kinexus Pro + rheometer. During scraping minimal pressure was applied to the agar surface to avoid chunks of agar detaching and contaminating the biofilm sample. The non-abrasive side of a Silicon carbide grinding paper (from Struers online store, Grit*#*2000) was attached to double sided sticky tape (3M 9084, Double Sided Paper Tape) and 20 mm holes were punched through. These circular adhesive backed waterproof grit papers were placed at the end of the rotating head and the bottom plate of the rheometer to reduce instances of slip between the plate and the biofilm sample. Zero gap configuration was established with the grit papers attached to both plates and then the biofilm sample was loaded onto the bottom stage. A constant normal force of 0.1*N* was applied and the sample was allowed to reach a steady state for at least 60*s*, which ensured uniform contact between the top plate and the biofilm sample. If required, any excess sample was trimmed to reduce instances of overfill. A solvent trap system was used to keep the samples hydrated for the duration of the measurements. For LAOS studies, the rheometer was operated in a strain-controlled mode and a 20 mm parallel plate geometry was used for our tests. A constant gap height was maintained for each run, which can vary between 0.5 and 1 mm due to the differences in biofilm volume harvested (The volume harvested per agar plate varies substantially for each of the tested species). After ensuring a constant gap height and a steady state normal force of 0.1*N* the biofilm sample was subjected to increasing values of oscillatory strain **γ** (0.3%, 0.5%, 0.8%, 1%, 3%, 5%, 8%, 10%, 25%, 50%, 75%, 100%, 200%, 400% and 500%) at a given frequency *ω* (0.5, 1, 2, 4 Hz). The raw values of angular displacement and torque were output from the Rspace software as a CSV file. The CSV files were read using MATLAB following which the waveforms were checked for stability and truncated to five cycles. The truncated waveforms were analysed using MITlaos software to get the LAOS measures. Standardised frequency and amplitude sweeps were performed on the sample to understand the material characteristics and map out the linear viscoelastic regime. All SAOS as well as LAOS experiments were repeated a minimum of five times. Experiments required for determining the slope of *I*_3_∕*I*_1_ vs. strain amplitude were performed once, since we had to sample finer values of strain amplitudes. To calculate the energy dissipation from the Lissajous bowditch plots, we used numerical integration in MATLAB. The endpoints of the averaged LB plots from MITlaos were joined to ensure that the curve is closed before performing the integration. The elastic Lissajous Bowditch plots can self-intersect, which can cause ambiguity in determination of area under the curve. To avoid ambiguities due to self-intersection, we use MATLAB code ‘intersections’ to check for points of intersection in the elastic LB plots. If self intersections are detected, the areas are segmented and total area is determined by summation of areas of each of the segments (Please see Supplementary Fig. 5, Supplementary note 3).

### Confocal microscopy

Following the growth of mat like biofilms on agar plates, 500 μL of 10 μM Syto 63 (S11345 from Thermo-fisher scientific) solution in Tris buffer was pipetted on the agar plate. The dye was allowed to stain the biofilms for 30 min. The biofilms were then scraped on the gene-frames (AB0576 from Thermo-fisher scientific) attached to a glass slide and sealed with a coverslip on the top. Images of the cellular structure were acquired with a Leica SP8 confocal microscope at a distance of 10–15 μm from the bottom glass coverslip using a ×100, 1.4 numerical aperture objective lens.

### Analysis of recorded waveforms using Chebyshev polynomial analysis

As the rheometer is operated in strain-controlled mode, the oscillatory strain imposed on the sample can be described as *γ* = *γ*_*o*_sin(*ωt*). The response of the material is assumed to be sinusoidal and can be written as *σ* = *σ*_*o*_sin(*ωt* + *δ*). Where, *γ*_*o*_ is the strain amplitude, *σ*_*o*_ is the magnitude of stress, *ω* is the oscillatory frequency of rheometer, *δ* is the phase angle between the input (strain) and output (stress) waveforms and t denotes time. Using the equations described in Ferry et al.^89^ and assuming that stress waveforms are sinusoidal (for various strain amplitudes), one can decompose the total stress into elastic and viscous components and calculate the elastic (*G'*) and viscous modulus (*G*″) of the material.

However, at large strain amplitudes the material enters the nonlinear regime and the stress waveform is not a simple sinusoid anymore. As a result, higher order harmonics need to be considered in order capture the true meaning of the waveform. The non-sinusoidal stress waveform can therefore be written in terms of Fourier expansion as:

1 $$\sigma (t;\omega ,{\gamma }_{0})\;=\;{\gamma }_{o}\sum _{n\;=\;1,3,....}^{}{G}_{n}^{\prime}(\omega ,{\gamma }_{o}){\mathrm{sin}}(n\omega t)\;+\;{G}_{n}^{{\prime\prime} }(\omega ,{\gamma }_{o}){\mathrm{cos}}(n\omega t)$$

where n represents the higher order harmonics. Only odd harmonics are considered because stress is assumed to bear odd symmetry with respect to shear strain or strain rate^49^. Ewoldt et al.^50^ proposed the use of orthogonal Chebyshev polynomials of first kind to approximate the nonlinear waveforms, as the higher order Chebyshev coefficients have physical meanings. Therefore Eq. (1) can be rewritten in terms of Chebyshev polynomials as:

2 $$\sigma (t;\omega ,{\gamma }_{0})\,=\,{\gamma }_{o}\sum _{n\;=\;1,3,....}^{}{e}_{n}(\omega ,{\gamma }_{o}){T}_{n}(x)\;+\;{\nu }_{n}(\omega ,{\gamma }_{o}){T}_{n}(y)$$

where, *e*_*n*_, *ν*_*n*_ are the n-th order elastic and viscous Chebyshev coefficents, *T*_*n*_ denotes the Chebyshev polynomial of first kind of n-th order, *x* = *γ*∕*γ*_*o*_ = sin(*ωt*) and $y=\dot{\gamma }/{\gamma }_{o}={\mathrm{cos}}(\omega t)$. Furthermore, by using the recursion identities of Chebyshev polynomials *T*_*n*_cos(*ωt*) = cos(*nωt*) and sin(*ωt*) = cos(*π*∕2 − *ωt*), which yields ${T}_{n}{\mathrm{sin}}(\omega t)={(-1)}^{\frac{n-1}{2}}{\mathrm{sin}}(n\omega t)$, one can directly express the Chebyshev coefficients in terms of the n-th order moduli:

3 $${e}_{n}\;=\;{G}_{n}^{\prime}{(-1)}^{\frac{(n-1)}{2}},{\nu }_{n}=\frac{{G}_{n}^{{\prime\prime} }}{\omega },n\in 1,3,.....$$

For *n* = 1, one can recover ${e}_{1}={G}_{1}^{\prime}$ and ${\nu }_{1}=\frac{{G}_{1}^{^{\prime\prime} }}{\omega }$. Traditionally, ${G}_{1}^{\prime} \sim {G}^{\prime}$ is known as the elastic modulus, ${G}_{1}^{{\prime\prime} }={\nu }_{1}\omega \sim {G}^{{\prime\prime} }$ is known as the viscous modulus and *ν*_1_ is the viscosity. The higher order coefficients can be related their respective moduli and in particular, the third order elastic (*e*_3_) and viscous (*ν*_3_) Chebyshev coefficients represent a physical meaning. A positive value, i.e. *e*_3_ > 0 and *ν*_3_ > 0 represents intracyle strain hardening and shear thickening, respectively; whereas negative values *e*_3_ < 0 and *ν*_3_ < 0 represents strain softening and shear thinning. These measures represent the a quantitative way of describing the elastic and viscous nonlinearities occurring in the material. One can also calculate the dimensionless strain stiffening index (*S*) and shear thickening index (*T*), which can be defined in terms of the higher order Chebyshev coefficients as follows:

4 $$S\;=\;\frac{4{e}_{3}....}{{e}_{1}\;+\;{e}_{3}\;+\;.....},T\;=\;\frac{4{e}_{3}...}{{\nu }_{1}\;+\;{\nu }_{3}\;+\;.....}$$

A more detailed description of the derivations of the above-mentioned equations and the physical interpretation can be found in the articles by Ewoldt et al.^50,90,91^.

### Reporting summary

Further information on research design is available in the Nature Research Reporting Summary linked to this article.

## Supplementary information

Supplementary Information

Reporting Summary

## Data Availability

All the raw data that were gathered can be found at the link: 10.6084/m9.figshare.11917890.

## Code availability

All the codes used to pre-process the raw data can be found at the link: 10.6084/m9.figshare.11917890. MITlaos software^91^ used to analyze the recorded waveforms can be requested from Professor Randy Ewoldt by emailing mitlaos@mit.edu.
