# Supplementary material for: Nonlinear rheological characteristics of single species bacterial biofilms
Source: NPJ Biofilms Microbiomes. 2020 Apr 14;6:19. doi: 10.1038/s41522-020-0126-1 (PMC7156450; doi:10.1038/s41522-020-0126-1)
Supplement: Supplementary file 1 — Supplementary Information [file 41522_2020_126_MOESM1_ESM.pdf]

Supplementary information: Nonlinear rheological characteristics of single species bacterial biofilms

Saikat Jana,[1,2,*] Samuel G.V. Charlton,[2] Lucy E. Eland,[3] J. Grant Burgess,[4] Anil Wipat,[3] Thomas P. Curtis,[2] and Jinju Chen[2,†]

[1]*School of Biomedical Sciences, University of Leeds, Leeds, United Kingdom*

[2]*School of Engineering, Newcastle University, Newcastle Upon Tyne, United Kingdom*

[3]*Interdisciplinary Computing & Complex BioSystems Research Group, School of Computing, Newcastle University, Newcastle upon Tyne, United Kingdom*

[4]*School of Natural & Environmental Sciences, Newcastle University, Newcastle upon Tyne, United Kingdom*

(Dated: March 7, 2020)

* saikatjana@gmail.com

† Jinju.Chen@newcastle.ac.uk

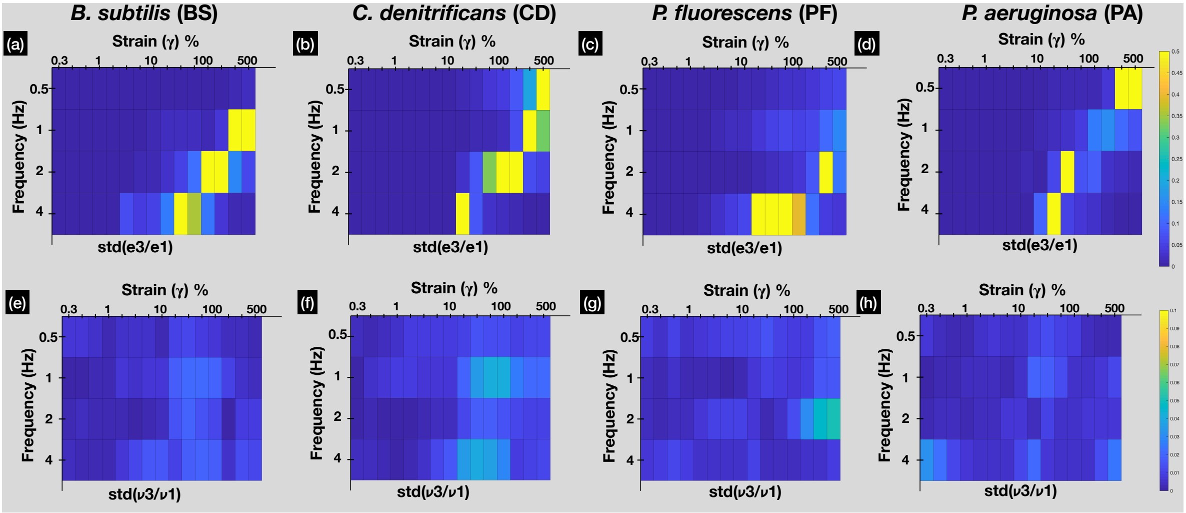

Supplementary figure 1. Figure showing the standard deviation of the elastic (e_3/e_1) and viscous (ν_3/ν_1) non-linearities in the Pipkin space for each of the bacteria species that were tested. Plots in the top row (a-d) show the standard deviation for the elastic nonlinearities at each point that was sampled in the Pipkin space. Plots in the bottom row (e-h) show the standard deviation for the viscous nonlinearities at each point that was sampled in the Pipkin space. Each of the point in the Pipkin space has a sample size of $n \geq 5$.

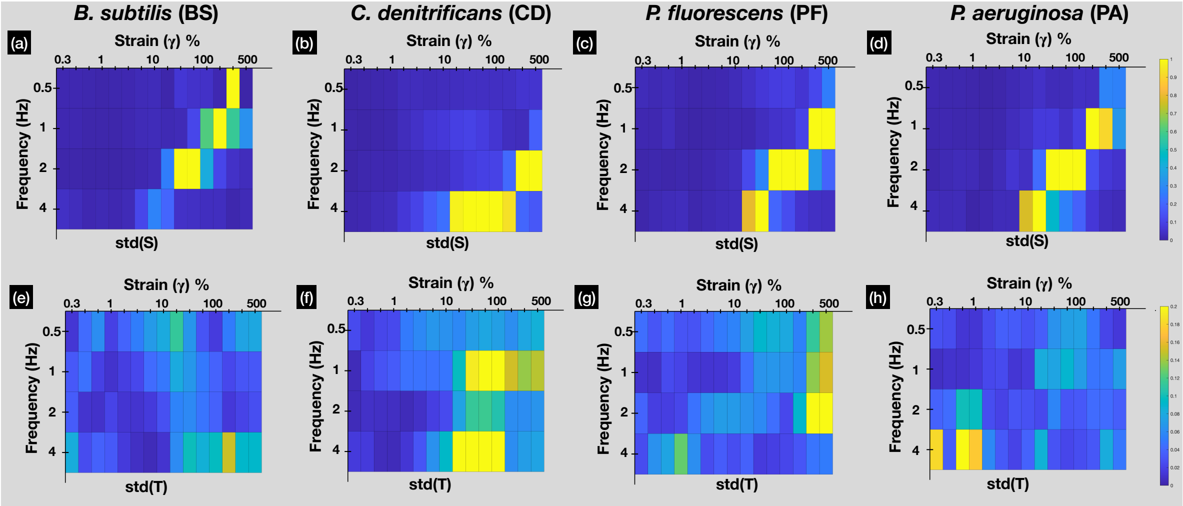

Supplementary figure 2. Figure showing the standard deviation of the Stiffening (S) and Thickening (T) indices in the Pipkin space for each of the bacteria species that were tested. Plots in the top row (a-d) show the standard deviation for S, at each point that was sampled in the Pipkin space. Plots in the bottom row (e-h) show the standard deviation for T, at each point that was sampled in the Pipkin space. Each of the point in the Pipkin space has a sample size of $n \geq 5$.

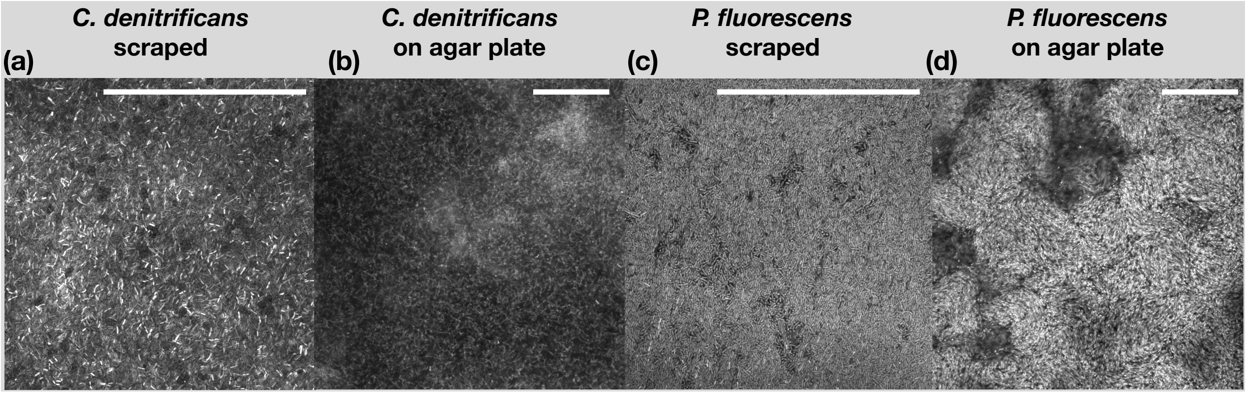

Supplementary figure 3. Shows the difference between scraped and natively imaged biofilms. (a) and (c) show the cellular structure of scraped biofilms. (b) and (d) show the cellular structure of biofilms grown on agar plate. All scale bars are 50 μm.

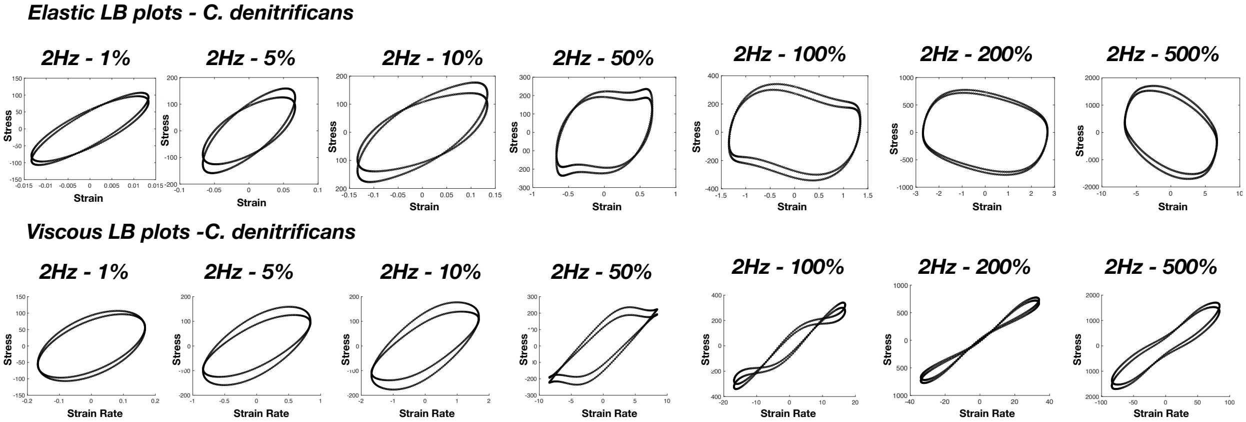

Supplementary figure 4. Shows the elastic and viscous Lissajous Bowditch plots for *C. denitrificans* biofilms subjected to two LAOS cycles with a recovery period of 15 minutes in between the two cycles.

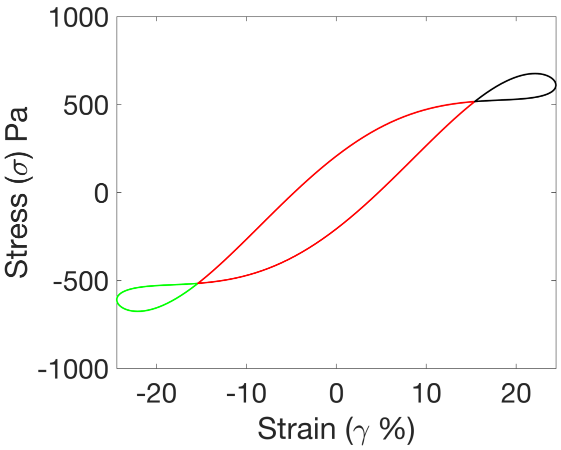

Supplementary figure 5. Figure shows a representative example of segmentation of a self intersecting elastic Lissajous Bowditch plots into three different self-enclosed curves (denoted by red, black and green curves).

Supplementary note 1. Confocal microscopy pictures of scraped and unscraped biofilms

The scraping procedure used to generate biofilm samples can introduce defects in otherwise continuous mats of biofilms that are grown on agar plates. To check for any defects or any disruption in structure we perform confocal imaging on biofilms grown on agar plates and compare them with confocal images of scraped biofilms. The cellular staining procedure is described within the methods section of the main text. After performing the cellular staining procedure, we rinse the biofilm three times with Tris buffer solution. The biofilm is subsequently imaged using a Spinning disk confocal microscope and a 60X water dipping lens with a numerical aperture of 1. This allows us to image native structure upto 30 μm deep from the top layer of the biofilm (beyond that depth the signal quality degrades). Owing to low numerical aperture of the water dipping lens the picture quality is relatively degraded (compared to the 100X, 1.4 numerical aperture lens used to image scraped biofilms), but none the less one is able to see the cellular structure of the biofilm in its native state.

Supplementary figure 3 (b) and (d) shows the cellular structure of *C. denitrificans* (CD) and *P. fluorescens* (PF) that were natively imaged on agar plate. For CD the disorder of the cellular structure can be seen both in the scraped and unscraped biofilm and existence of small void spaces can be observed. In the case of PF, natively grown biofilms on agar show some local alignment and large void spaces are observed because parts of the biofilm are not in the focal plane. For scraped PF, small void spaces are observed and local ordering of cells are not as prominent as in the unscraped biofilm.

Supplementary note 2. Does biofilm viscoelasticity recover even after application of extremely large strain?

We present Lissajous-bowditch (LB) plots for CD biofilms that are subjected to two successive LAOS cycles with 15 minute recovery time in between the runs in supplementary figure 4. The LB plots are very similar in both the runs indicating similarity of viscoelastic measures in both the runs, perhaps suggesting that the biofilms recover. However, more comprehensive investigations are needed to establish the thixotropic nature of biofilms; as it is expected to be dependent on the polymeric composition and eventually the species of microbes that form them.

Supplementary note 3. Segmentation of self-intersecting Lissajous Bowditch plots

To remove the ambiguity in calculating the the area under the curve for self intersecting Lissajous Bowditch plots. We now incorporate an additional check for self-intersection in Lissajous Bowditch plots in our code, using the MATLAB subroutine 'intersections'. If self-intersections are detected, which is usually a pair of points; we split the curves into set of three self-enclosed curves (see supplementary figure 5). The areas enclosed by each of the three curves is calculated numerically and added to get the total area.
